# Assessment of coronary artery intimal thickening in patients with a previous diagnosis of Kawasaki disease by using high resolution transthoracic echocardiography: our experience

**DOI:** 10.1186/1471-2261-14-106

**Published:** 2014-08-20

**Authors:** Valentina Giacchi, Pietro Sciacca, Ileana Stella, Martina Filippelli, Patrizia Barone, Mario La Rosa, Salvatore Leonardi

**Affiliations:** 1 Pediatric Pneumo-Allergology and Cystic Fibrosis Department, Pediatric Cardiology Clinic, AOU “Policlinico-Vittorio Emanuele”, University of Catania, Catania, Italy

**Keywords:** Kawasaki disease, High resolution transthoracic echocardiography, Cardiovascular risk, Coronary intimal thickening

## Abstract

**Background:**

Kawasaki disease (KD) is a generalized systemic vasculitis of unknown etiology involving medium and small size blood vessels, particularly the coronary arteries. In these vessels a progressive stenosis may result from active remodeling with an intimal proliferation and neoangiogenesis. The aim of our study was to assess, by using high-resolution transthoracic 2D Echocardiography, if subjects with a previous diagnosis of Kawasaki disease after several years show a coronary intimal thickening, suggestive of a persistent cardiovascular risk.

**Methods:**

We assessed measurement of thickening, inner diameter and outer diameter of coronary arteries using 2D Echocardiography (Philips E 33 with multy-frequency S8-3 and S12-4 probes) and examining the proximal portion of left main coronary artery just above the aortic valve with parasternal short axis view.

**Results:**

We found a significant intimal thickening in patients with previous Kawasaki disease compared to healthy controls. In particular, we noticed that also subjects not suffering from coronary impairment in acute phase have higher values of thickening than healthy controls, and this wall thickening may confer a higher cardiovascular risk.

**Conclusions:**

Therefore we concluded that the assessment of coronary artery thickening by high-resolution transthoracic 2D Echocardiography may become an essential instrument to evaluate late cardiovascular risk in subjects with a diagnosis of Kawasaki disease in childhood.

## Background

Kawasaki disease (KD) is a systemic vasculitis affecting infants and young children [1,2] which may potentially involve heart [3], small and medium-size arteries, and can lead to the formation of aneurysms, especially in the coronary arteries [4,5].

During the acute phase of the disease, mortality peaks from 15 to 45 days after the onset of fever, usually due to cardiac sequelae such as arrhythmia and myocardial infarction (MI) [5,6]. It has been noticed that sudden death from myocardial infarction may also occur after many years in individuals who had previously developed coronary artery aneurysms (CAA) and stenosis, and it seems today that several cases of fatal and nonfatal MIs in young adults have been attributed to “missed” KD diagnosis in childhood [5].

Regarding the pathogenesis of vascular damage, it is well-known that in Kawasaki disease an arterial active remodeling occurs due to intimal proliferation and neoangiogenesis; the intima, rich in smooth muscle cells, and fibrous layers, appears markedly thickened with linearly-arranged microvessels [6].

It has lately been noticed that the high resolution transthoracic echocardiography is able to detect intimal thickening in the wall of left main and left anterior descending (LAD) coronary arteries in adults with subclinical atherosclerosis [7,8]. We hypothesized that this technique, applied to the study of proximal left main coronary artery, could be useful in monitoring the follow-up of KD after the acute phase.

The aim of the present study was to assess coronary artery intimal thickening by using high resolution transthoracic 2D Echocardiography in patients with a previous diagnosis of Kawasaki disease several years after the acute event.

## Methods

We conducted an observational cross-sectional case–control double-blind study on 31 patients with a previous diagnosis of KD who had been hospitalized in childhood since January 1990 to December 2000 in our Pediatric Department of the University of Catania, Italy.

Every patient if of age, or at least one parent or legal guardian if underage, gave their written informed consent before the patient’s inclusion in the study. The study was conducted in accordance with the Helsinki Declaration, and the study protocol was approved by the (local) Ethics Committee of the Medical University of Catania.

Five patients were omitted from the study as they were current smokers. In the same way we recruited 26 voluntary healthy subjects among students of school and University to be used as controls.

The main features of the 26 patients at the moment of diagnosis are reported in Table 1.

**Table 1:** Features of 26 patients at diagnosis

**Gender**	**Clinical form**	**ECG**	**Cardiac manifestations**	**Risk class**	**Therapy**	**Age at diagnosis**
M:18 (69.2%)	T: 14 (53.8%)	N: 12 (46.2%)	NCI: 14 (54%)	I: 20 (76.9%)	ASA + IVIG 20 (76.9%)	< 6 ms: 2 (7.7%)
F:8 (30.8%)	I: 9 (34.6%)	SST: 9 (34.6%)	TAC: 3 (11.5%)	II: 3 (11.5%)	ASA 3 (11.5%)	6 ms- 5 ys: 21 (80.8%)
	A: 3 (11.5%)	PRBBB: 3 (11.5%)	PAC: 3 (11.5%)	III: 2 (7.7%)	IVIG 2 (7.7%)	> 5 ys:3 (11.5%)
		NSDVR: 2 (7.7%)	PE: 3 (11.5%)	IV: 1 (3.9%)	No therapy 1 (3.9%)	
			MR: 3 (11.5%)			

*Abbreviations: M* male, *F* female, *T* Typical, *I* Incomplete, *A* Atypical, *N* normal, *SST* slight sinus tachycardia, *PRBBB* partial right bundle branch block, *NSDVR* non-specific disorders of ventricular repolarization, *NCI* no cardiac impairment, *TAC* transient anomalies of coronaries, *PAC* persistent anomalies of coronaries, *PE* pericardial effusion, *MR* mitral regurgitation.

The diagnosis of Kawasaki disease was performed according to the criteria of the American Heart Association (AHA) Committee on Rheumatic fever, Endocarditis and Kawasaki disease [9,10]: a typical form of Kawasaki disease was detected in 14 patients (53.3%), an incomplete form in 9 (34.6%) and an atypical form in 3 (11.5%).

Every patient was assigned to corresponding risk class: 20 (76.9%) to the first risk class, 3 (11.5%) to the second, 2 (7.7%) to the third and 1 (3.9%) to the fourth. All patients belonging to third and fourth risk classes were affected by typical form, whereas patients of first and second classes presented typical, atypical or incomplete form.

According to the guidelines at the time of diagnosis, 20/26 (76.9%) had been on both acetylsalicylic acid and intravenous immunoglobulin (IVIG) therapy, (400–500 mg/Kg/day for 4–5 consecutive days or 1–2 g/Kg in a single dose), 3/26 (11.5%) only on acetylsalicylic acid therapy, 2/26 (7.7%) only on IVIG therapy, whereas in a single case (3.9%) the parents refused therapy.

At present, the age ranges from 4 to 27, with an average of 13.3 ± 7.4 and a time from the beginning of the illness ranging from 3 to 22 years.

We performed Electrocardiogram and 2D Echocardiography in all patients.

The laboratory and cardiologic data were compared with those of the 26 healthy subjects of the same age.

The same experienced echocardiographer, unaware of whether he was dealing with a case or a control, assessed measurement of thickening, inner diameter and outer diameter of coronary arteries using 2D Echocardiography (Philips E 33 with multy-frequency S8-3 and S12-4 probes, using the setting scheduled by the echo machine) and examining the proximal portion of left main coronary artery just above the aortic valve with parasternal short axis view. Echocardiographic recordings of four separate cardiac cycles were captured by zoom image. Optimal gain setting was adjusted for maximal delineating the outer edge from the inner edge of the line representing the vascular wall. The thicker walled vascular structure was identified as multiple linear echoes which branched off just above from the aortic valve and was measured edge to edge at diastolic period.

### Statistical analysis

We calculated means, standard deviation, standard error (SE) and Median for all variables. We adjusted values of coronary thickening for BSA (Body Surface Area) and performed t-student and Mann–Whitney test to compare these values between cases and controls.

*P* value < 0.05 was considered statistically significant.

## Results

We have not found any significant difference in subjective and laboratory parameters between patients and healthy controls Table 2. Both cases and controls presented normal values of cholesterol, Body Mass Index (BMI), blood pressure and nobody was alcohol drinker or had any other risk factor for atherosclerotic disease.

**Table 2:** Subjective and laboratory parameters in cases and healthy controls

***Variables***	***Cases***	***Controls***	**P value**
Age (years)	13.3 ± 7,4	12.6 ± 7,4	ns
Male	69%	73%	ns
Female	31%	27%	ns
BMI	20.4 ± 3.9	20.6 ± 2.8	ns
BSA (m^2^)	1.33 ± 0.44	1.36 ± 0.40	ns
Total cholesterol (mg/dl)	177 ± 22	170 ± 36	ns
Triglycerides (mg/dl)	61 ± 29	65 ± 24	ns
Low density lipoprotein (mg/dl)	110 ± 19	110 ± 18	ns
C-reactive protein > 0,10 (mg/dl)	11.5%	7.7%	ns
Platelets (x10^3^)/mmc	309 ± 93	359 ± 114	ns
Systolic blood pressure (mmHg)	110 ± 14.4	108 ± 12	ns
Diastolic blood pressure (mmHg)	64 ± 9.8	63 ± 11	ns

The Electrocardiogram did not show any signs of ischemia in either cases and controls.

The 2D Echocardiographic assessment of systolic and diastolic function showed normal values in both patients and healthy controls.

We confirmed persistent anomalies of coronary arteries already described at diagnosis in two of the patients (one patient with a giant aneurism of left coronary artery and another one with two aneurisms in the left and right coronary arteries respectively), both with previous diagnosis of typical KD, assigned to 3 and 4 risk class respectively.

Regarding intimal thickness, we detected significant higher thickening values adjusted for BSA in cases than healthy controls (5.3 mm ± 4.4, Median 3.7, SE 0.8, versus 2.7 mm ± 1.2, Median 2.6, SE 0.2, p <0.01) Table 3.

**Table 3:** Thickening and thickening adjusted for BSA in cases and healthy controls

**Parameter**	**Cases**	**Controls**	**P Value**
**Thickening (mm)**	3.5 ± 2.2	1.9 ± 0.6	**< 0.01**
**Thickening adjusted for BSA (mm)**	5.3 ± 4.4	2.7 ± 1.2	**< 0.01**

Assessing the main values of thickening adjusted for BSA in the different risk classes, we found values equal to 4.5 mm ± 3.7, Median 3.4, SE 0.8 in the risk class 1 and, assembling patients belonging to risk classes 2-3-4, we detected values equal to 7.9 mm ± 5.8, Median 6.0, SE 2.4 in the risk class 2-3-4 group. We noticed significant difference in thickening values between controls and patients belonging to risk class 1 and in controls and patients belonging to risk class 2-3-4 group (p < 0.05) but not between patients belonging to risk class 1 and patients belonging to risk class 2-3-4 group (p = ns) Table 4.

**Table 4:** Thickening and thickening adjusted for BSA in controls, patients belonging to risk class 1 and patients belonging to risk class 2-3-4 group

**Category**	**Thickening (mm)**	**Thickening adjusted for BSA (mm)**
**A (Controls)**	1.9 ± 0.6	2.7 ± 1.2
**B (Risk class 1)**	3.0 ± 1.7	4.5 ± 3.7
**C (Risk class 2-3-4)**	5.3 ± 3.1	7.9 ± 5.8

**Thickening:** A versus B: p < 0.01; A versus C:p < 0.01; B versus C: NS; **Thickening adjusted for BSA:** A versus B: p < 0.05; A versus C:p < 0.05; B versus C: NS.

## Discussion

Our study might provide new data in the long-term follow-up of Kawasaki disease since our patients showed a significant thickening of coronary intimal wall not related to the severity of the Kawasaki disease during the acute phase. Coronary intimal alteration could indirectly mean a higher cardiovascular risk.

It has been noticed that in coronary atherosclerosis, a diffuse disease process that rarely spares the proximal coronary arteries, a severe intimal thickening may be assessed as a site of localized stenosis. Accurate baseline measurements of the luminal and external diameters and wall thickness of proximal and mid LAD coronary artery have proved to be obtainable by using the high resolution transthoracic echocardiography techniques [11,12].

We successfully applied the same method to the proximal left main coronary artery: by using high resolution transthoracic probes we detected in morphology of proximal left main coronary artery structural features not so far different from atherosclerosis-induced positive remodeling, already showed in other coronary vascular territories of patients with confirmed significant luminal coronary artery disease [13].

In fact studies prior to IVIG therapy showed that a potential mechanism of coronary artery occlusion in long-term KD could be represented by the progression of the thickening of intimal layer and particularly, but not only, in the aneurysm inlet or outlet which is often associated to calcification [14,15]. This, with any combination of thrombus formation [16] might lead to acute myocardial infarction in young adults with sequelae of KD after several years [17].

Afterwards, Takahashi et al. histologically examined twenty-four arteries of six autopsy cases of patients older than 15 years with coronary arterial lesions caused by arteritis in childhood and detected in both patients without aneurysms and those with manifest recanalized lumens after thrombotic occlusion of the aneurysms “new intimal thickening” in addition to the preexisting intimal thickening caused by arteritis in the acute phase of KD. They concluded that subjects with a history of KD present a risk factor for atherosclerosis later in life [18].

Iemura et al. found various degrees of intimal thickening but normal media on intravascular ultrasound imaging of the sites of regressed aneurysms. These intravascular ultrasound findings were similar to those in arteriosclerosis [19].

Intimal thickening, detectable in proximal left main coronary artery, occurs as a result of augmented vascular smooth muscle cell proliferation, in addition to increased vascular smooth muscle cell migration, extracellular matrix synthesis and phenotypic change [20,21].

It has recently been proposed that the Wnt/β-catenin pathway, involved in the regulation of embryogenesis and development, but also in cell proliferation, differentiation, polarity, migration, and invasion [22], plays a role in vascular smooth muscle cell proliferation and thereby intimal thickening [23-29].

**Figure 1:**
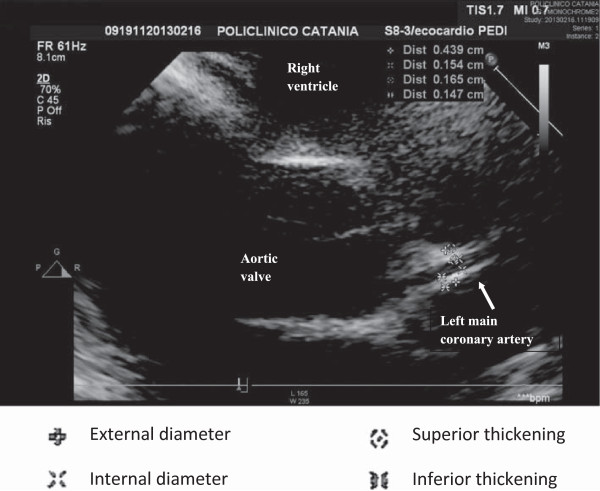
**Abnormal left coronary artery.** Legend:  External diameter;  Internal diameter;  Superior thickening;  Inferior thickening.

**Figure 2:**
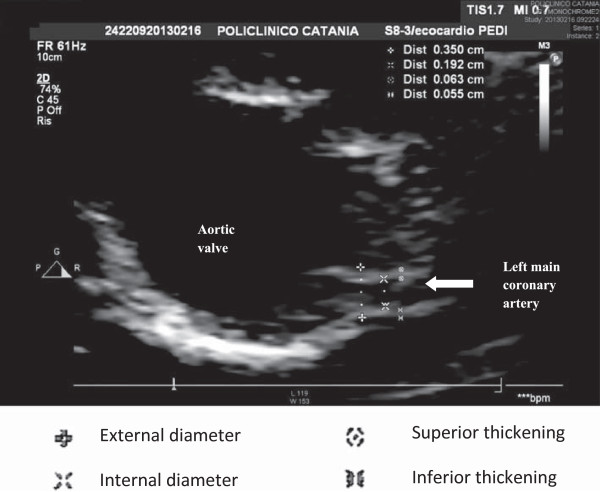
**Normal left coronary artery.** Legend:  External diameter;  Internal diameter;  Superior thickening;  Inferior thickening.

Therefore, the risk for later KD complications suggests the necessity of a long-term follow-up of KD patients beyond childhood years.We showed that the left main coronary artery wall thickness of subjects with previous Kawasaki disease were significantly larger (Figure 1) than those of healthy subjects (Figure 2) such as indicating atherosclerotic disease. The inner diameters, instead, were normal in both controls and cases, also in those with previous transient aneurysms suggesting, as an explanation, the phenomenon of positive remodeling.

## Conclusion

Our study shows the high sensibility of high resolution transthoracic 2D Echocardiography to the detection of minimal alterations in coronary wall due to intimal thickening in patients with previous Kawasaki disease, also if studied several years after acute phase. In our experience, all patients with a previous diagnosis of Kawasaki disease, independently from the risk class, present a significant thickening of coronary arteries if compared to healthy controls. We state that this may result in premature atherosclerotic cardiovascular disease and therefore the measurement of coronary intimal thickening by high-resolution transthoracic 2D Echocardiography should be added to the follow-up protocol of Kawasaki disease because of its uninvasiveness and reproducibility.

Further investigation is obviously requested to confirm our data. We suggest that this be studied in a larger, multi-institutional study.

## Abbreviations

KD: Kawasaki disease; MI: Myocardial infarction; CAA: Coronary artery aneurysms; LAD: Left anterior descending; IVIG: Intravenous immunoglobulin; SE: Standard error; BSA: Body Surface Area; BMI: Body Mass Index.

## Competing interests

The authors declare that they have no competing interests.

## Authors’ contributions

VG carried out the collection and assembly of data, the analysis and interpretation of data, the draft of the manuscript. PS conceived of the study and participated in the design and coordination, helped to draft the manuscript and gave the final approvation. IS participated in the collection, assembly, analysis and interpretation of data, MF and PB participated in collection and assembling of data. MLR participated in the critical revision of the manuscript. SL performed the critical revision of the manuscript and gave the final approval. All authors read and approved the final manuscript.

## Pre-publication history

The pre-publication history for this paper can be accessed here:

http://www.biomedcentral.com/1471-2261/14/106/prepub
